# Two stents’ dislodgement in the left main coronary artery: a case report

**DOI:** 10.1186/s13256-024-04491-6

**Published:** 2024-03-18

**Authors:** Salim Arous, Hatim Zahidi, Mohamed El Ghali Benouna, Rachida Habbal

**Affiliations:** Cardiology Department, Ibn Rochd University Hospital, Casablanca, Morocco

**Keywords:** Left main stenting, Stent dislodgement, Percutaneous intervention, Complications

## Abstract

**Background:**

Stent dislodgement is a life-threatening complication that can result in coronary artery embolization, stent thrombosis, acute myocardial infarction, and even death. Severely angulated, heavily calcified, and previously stented coronary arteries are associated risk factors. With the development of different lesion preparation techniques and the drug eluting stent era, the reported incidence of stent dislodgement has decreased to < 1% in the last few years.

**Case presentation:**

We report a case of a 64-year-old Moroccan man complicated during percutaneous intervention in the left main artery by the loss of two stents. This complication was successfully managed by passing the stent’s balloon into the stent and then fully expanding it. In our case, the device’s characteristics were involved and could play a role in such complications, but it is still not well understood.

**Conclusions:**

The main treatment option is stent retrieval with different available techniques. If retrieval of the stent is impossible, crushing it against the blood vessel wall could be considered.

## Background

Stent dislodgement is a challenging and serious complication during percutaneous intervention (PCI). It is a life-threatening complication that can result in coronary artery embolization, stent thrombosis, acute myocardial infarction, and even death. Moreover, if a lost stent moves outside of the coronary artery, it may cause cerebral stroke, and peripheral artery occlusion [1].

Several lesion characteristics are related to stent dislodgement, such as heavily calcified stenosis, severe tortuosity, long diffuse lesions, and previously implanted stents. Other stent-related factors may be involved such as stent designs, stent strut thickness, and metal platforms [2]. With the development of different lesion preparation techniques and the drug eluting stent era, the reported incidence of stent dislodgement has decreased to < 1% in the last few years [3]. But unfortunately, it still occurs; therefore, interventional cardiologists must be familiar with common stent retrieval techniques.

We report a complicated case of the loss of two stent that occurred during PCI in the left main coronary artery, and also demonstrate a safe and successful approach to deal with this issue.

## Case presentation

A 64-year-old Moroccan man with a medical history of an acute coronary syndrome 2 months previous leading to angioplasty of the mid and distal left anterior descending artery (LAD) with two drug-eluting stents. The previous coronarography showed a distal left main stenosis. After medical surgical discussion and considering the low syntax score with no diabetes, the patient was admitted in the cathlab for a scheduled distal left main angioplasty. The patient had persistent Canadian class III angina on optimal medical therapy. He was hemodynamically stable on physical examination with a blood pressure at 128/72 mmHg, a pulse at 90 beats per minute, and saturation at 96% on room air. The physical exam was normal. The lungs were clear to auscultation, there was no leg edema, and peripheral pulses were palpable. Electrocardiogram showed a regular sinus rhythm with anterior Q waves. The troponin level and usual biological assessment was normal. Echocardiography found features of ischemic heart disease with anterior and apical hypokinetic wall motion and a preserved left ventricular ejection fraction at 50%, normal left ventricle feeling pressure, and no mitral regurgitation or pulmonary hypertension.

**Fig. 1:**
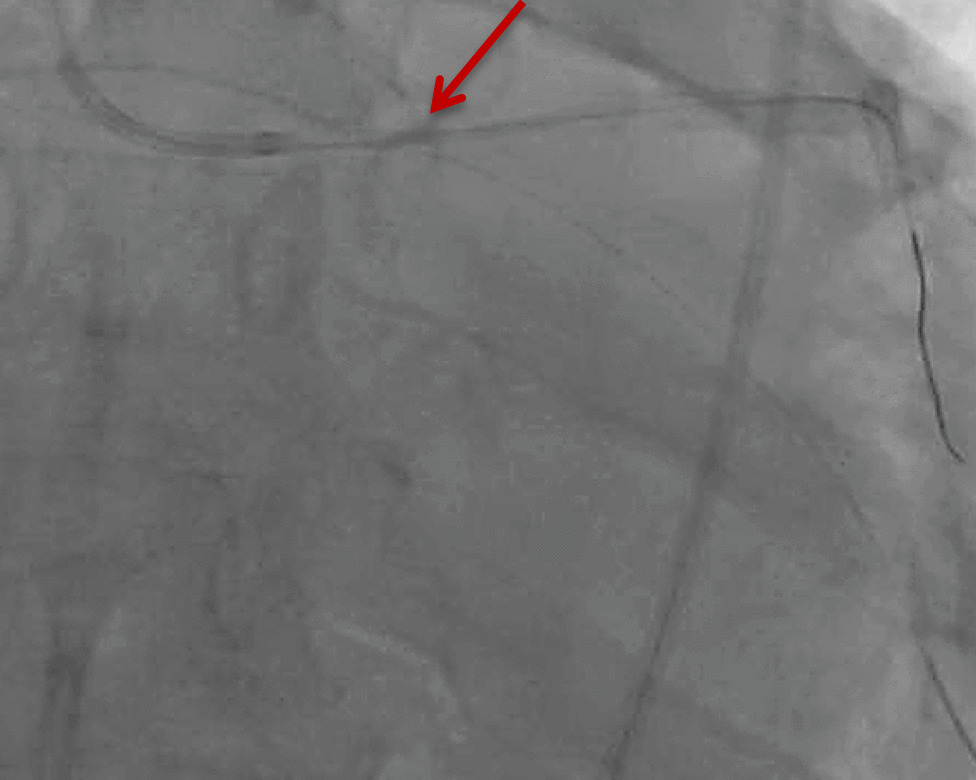
Stent loss in the proximal LAD (red arrow)

The patient underwent angioplasty via the right femoral artery through 7-French femoral sheath. There was a 70-90% stenosis of the distal left main (LM) coronary artery including proximal LAD and circumflex (LCx; MEDINA 1.1.1). Using a 7 French extra back up guiding catheter well engaged in the left main, both LAD and LCx were wired with workhorse wires Sion blue and BMW universal II respectively. PCI was attempted from the left main to the proximal stenosis of LAD initially with a 4 × 26 mm Orsiro Biotronik Drug-Eluting Stent (DES). The stent was dislodged just after the exit of the guiding catheter with guidewire still maintained in the LAD (Fig. 1).

**Fig. 2:**
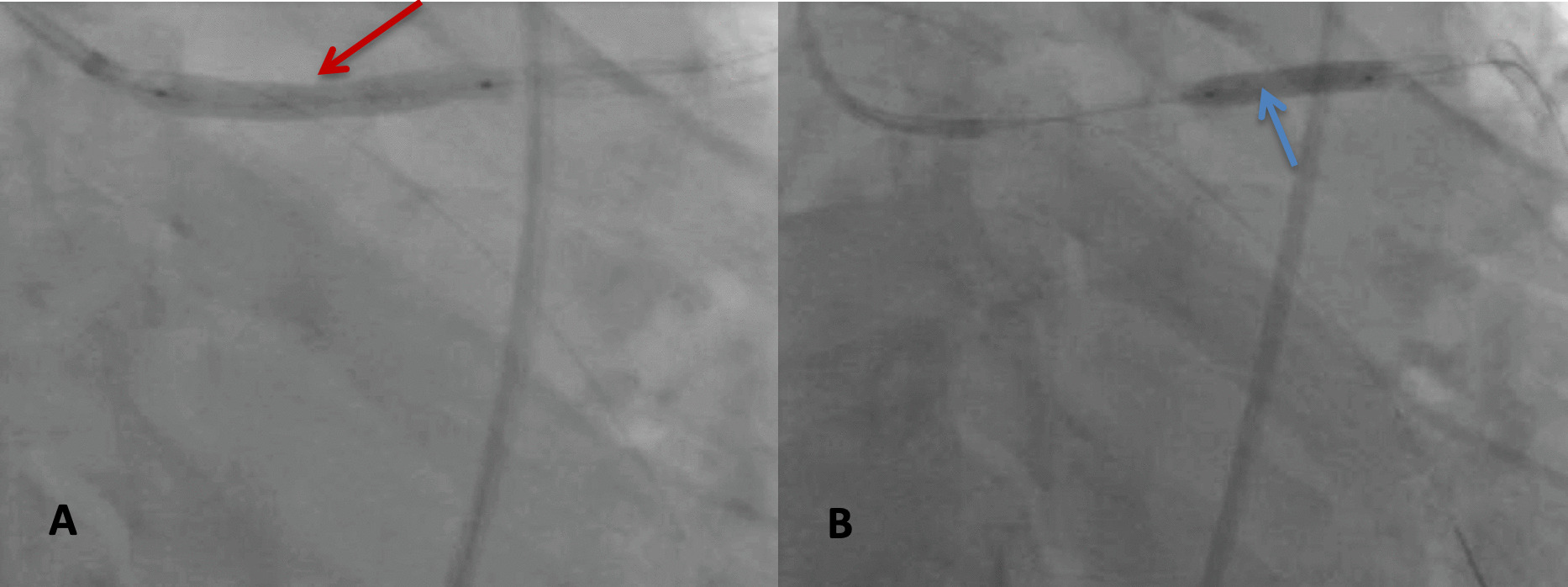
**A** Successful deployment of proximal and middle part of the stent of proximal LAD (red arrow), **B** Post-dilatation of the distal part of the stent to ensure a full expansion (blue arrow)

Since the guidewire position was maintained through the lost stent, an attempt to advance the stent’s balloon through the lost stent was performed. The balloon could pass, and about 70% of the dislodged stent was deployed (Fig. 2A). Other small balloons were used to expand the distal part of the stent. After post-dilatation with a final balloon 4.00 × 15 mm, the whole stent was successfully deployed and covered 4 mm in the distal left main to proximal LAD (Fig. 2B).

**Fig. 3:**
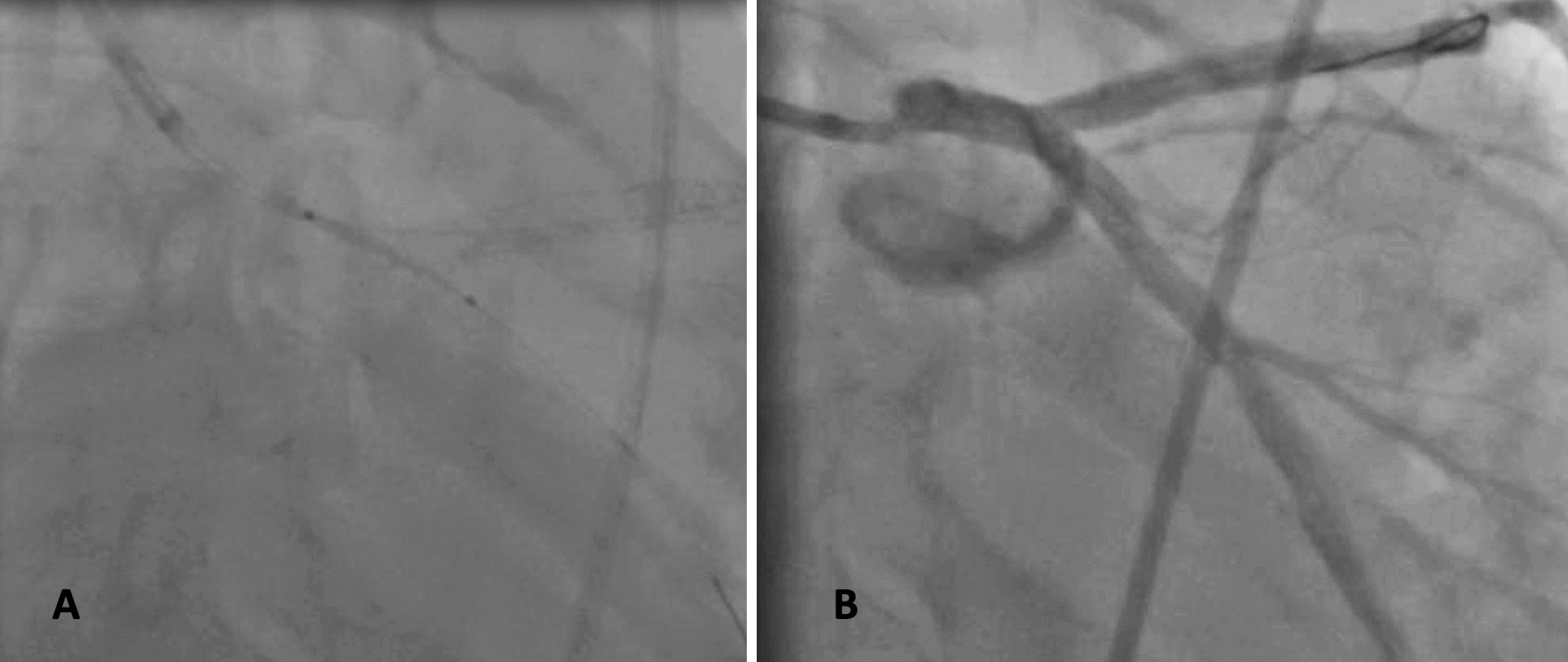
**A** Partial stent loss in the left main–ostial circumflex, **B** Final result after stenting the ostial circumflex

**Fig. 4:**
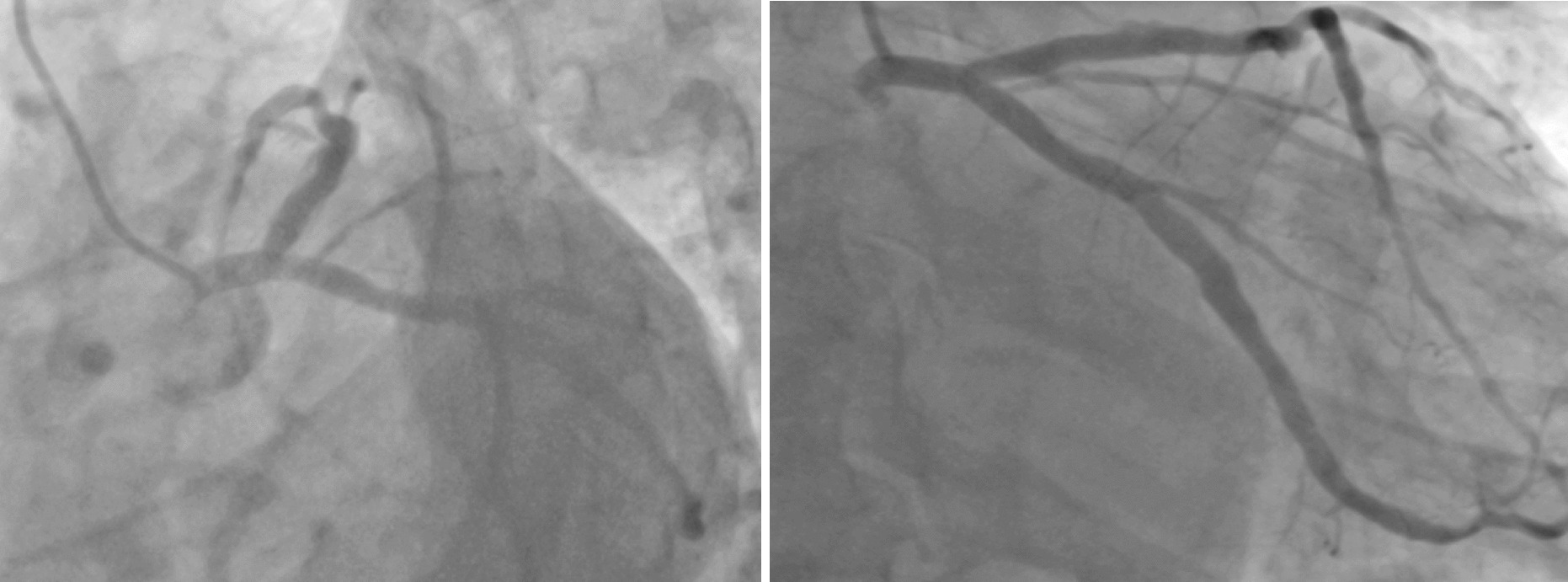
Angiogram at 7 months showing a good result with no significant restenosis

The guide wires were switched to ensure that they were not twisted. The LCx was predilated with a 3.00 × 15 mm compliant balloon. Then another Orsiro Biotronik 4.0 × 22 mm was chosen to treat the left main–circumflex stenosis. But similarly to the first stent, at the exit of the guide catheter, it was partially dislodged in the left main–circumflex (Fig. 3A). The attempt to retrieve the stent was unsuccessful, and then the stent was deployed in the left main after advancing the stent’s balloon. But unfortunately, the stent failed to completely cross and cover the circumflex lesion. The proximal circumflex was then treated with a 4.00 × 15 mm Promus Elite Boston stent without any issue. A 3.00 × 15 mm compliant balloon was used to open struts in the left main–circumflex, then full stent expansion was obtained by a final proximal optimization technique (POT). The final angiography showed a fairly good result with no residual stenosis (Fig. 3B). The patient was regularly followed up with complete resolution of chest pain. A systematic angiogram was performed 7 months after, which found a satisfying result with no restenosis (Fig. 4). Stent boost and intravascular ultrasound (IVUS) were not available.

## Discussion

The incidence of stent loss has significantly declined in recent years despite the high number of complex PCI procedures performed. The incidence of stent loss in studies published before 2000, between 2000 and 2005, and after 2005 was 5%, 1%, and 0.3%, respectively, as shown in Table 1 [4–7]. This may reflect the continuous improvement of stent technology, including pre-mounted stents, better cross-sectional profiles, and improved stent delivery systems, as well as the increasing experience of operators.

**Table 1:** Decrease in incidence of stent loss in different studies between 1998 and 2020

Author	Year	Incidence of stent loss
Cantor *et al*. [5]	1998	108/1303 (8.3%)
Eggebrecht *et al*. [6]	2000	20/2211 (0.9%)
Brilakis *et al*. [4]	2004	38/11,773 (0.32%)
Rigatelli *et al*. [7]	2020	144/25,962 (0.56%)

Several risk factors for stent loss have been proposed by previous studies. As reported by Laarman *et al*., direct stenting may be associated with a higher risk of stent loss compared with a strategy of pre-dilation, presumably due to the increased resistance to stent advancement through the lesion [8]. The other most common causes of stent dislodgement are attempting to deliver a stent through a previously deployed stent. Stenting from distal to proximal may prevent such a complication. Sometimes, stent deformation can occur during attempts to cross the lesion, and then the stent is stripped off while attempting to withdraw it into the guide catheter [9, 10]. Tortuous vessels and heavily calcified lesions are other factors that could contribute to stent loss [11, 12]. However, the development of preparation lesion techniques (such as predilation, orbital or rotational atherectomy, and shockwave) has allowed for better stent delivery, and therefore lowered the risk of stent loss. In fact, adequate and careful preparation of the stenting site, such as the reduction of calcium burden, intuitively reduces the risk of stent decrimping, dislodgement, or embolization. In our patient, the lesion was not heavily calcified or very tight; that is why a direct stenting approach was chosen and justified. Moreover, the guide catheter was well engaged in the left main with no guide wire twist, and the stent loss happened just after the exit from the guide catheter and not in the attempt to cross the lesion. In this case, a lack of lesion preparation may play a role, but since the stent dislodgement happened just at the exit of the guide catheter and not in the attempt to cross the lesion, stent-related factors may be involved in the stent dislodgement in our case.

Latest reports suggest that other factors related to the stent may increase the risk of stent dislodgement. Kyoung Woo Seo and his team compared dislodgement forces in five different types of DES: Firehawk, Xience Sierra, Orsiro, Resolute Onyx, and Synergy. They found that the peak dislodgement force was significantly lower in the Orsiro than in all the other stents. Moreover, during the pullback of the stents, other DESs except the Orsiro were not removed from the delivery system despite the stents being broken. The Orsiro stent was easily removed if a strut was dislodged from the balloon [13]. Rigatelli *et al*. reported on the relationship between stent strut thickness and dislodgement. They divided the stents into thick (> 81 µm strut thickness) and ultrathin (≤ 81 µm strut thickness) strut stent groups. Stent dislodgement is more common in ultrathin than in thick strut stents (0.28% versus 0.78%, *P* < 0.001). The ultrathin strut group included Resolute Onyx, Orsiro, Xience, and Coroflex. Although they did not report the incidence of each stent, approximately half of the stents were Orsiro stents, which had the thinnest strut, and this group displayed a higher rate of stent dislodgement [7, 14]. In the same report, the authors explain that, even if the rate of dislodgement is higher with ultrathin stents, they could be easily parked or crushed with a lower risk of stent thrombosis. This may suggest that the use of a thin strut DES in our patient could have played a role in stent dislodgement. Moreover, the use of another thick strut stent without any problem in our case supports these findings.

Few systematic studies have been conducted on the incidence and outcomes of stent dislodgement in patients treated with DES over the last 20 years. In 2007 Yucel Colkesen *et al*. observed a 0.29% incidence of stent embolization in a series of 4797 patients that resulted in bypass surgery in most cases [15]. Alomar *et al*., in a meta-analysis across the 1991–2012 period, reported stent loss in 1.3% of 71,655 PCI, resulting in a 19% complication rate including coronary artery bypass graft surgery, 18% for myocardial infarction, a 19% death rate, 6% for bleeding requiring transfusion, 3% for vascular access complications, 0.6% rate of cerebrovascular accident [3]. These complications appear to be less frequent in a recent report from 2016 where the incidence of stent loss was only 0.56% out of 25,692 PCI. Concerning complications, there was no acute death or need for urgent coronary artery bypass graft (CABG), and they were all managed, including through vessel dissection, coronary perforation, stent thrombosis, and acute myocardial infarction [7].

Appropriate management of the dislodged stent is important to prevent those complications. Several management options are available for dealing with this critical situation and include: (1) the small-balloon technique—only feasible if the guidewire position is maintained through the lost stent—which is a simple technique in which a small balloon is advanced through the lost stent, inflated distally, and withdrawn, displacing the lost stent into the guiding catheter [6]; (2) retrieving the stent with snare; (3) leaving the stent in the coronary vessel and crushing it with another stent [16, 17]; and (4) parking the stent in a peripheral vessel in the case of extra-coronary migration [18, 19]. Actually, most of the cases are successfully managed via a percutaneous approach, while surgical intervention is required mainly if the stent embolizes and causes coronary occlusion leading to hemodynamic instability. However, peripheral stent loss rarely causes complications, and in many cases, it cannot be localized. In our case, since we could maintain the guidewire position through the lost stent, we could repass the stent balloon and deploy and fully expand the stent.

## Conclusions

Stent dislodgement is a rare but serious complication. Severely angulated, heavily calcified, and previously stented coronary arteries are associated risk factors. It is probable that the device’s characteristics could play a role in such complications, but it is still not well understood. The main treatment option is stent retrieval with different available techniques. If retrieval of the stent is impossible, crushing it against the blood vessel wall could be considered.

## Data Availability

The published information is available from the corresponding author on reasonable request.
